# C. Hering

**Published:** 1881-01

**Authors:** Edward Bayard

**Affiliations:** New York


### CONSTANTINE HERING.

BY EDWARD BAYARD, M. D., NEW YORK.

If a great man is one to whom God has given large gifts, and who has cultivated them to the extent of his powers for the best interests of his fellow beings, then Constantine Hering was a great man. He was not a money getter. His powers did not work in the direction of accumulating property. He did not care to amass this world's goods; but he wanted to be rich in learning, especially in all that pertained to his profession.

He was logical, discriminating, a great lover of nature and a close observer of her. He was a hard student, of unwearied industry. He "sought truth earnestly, and he found it." He made note of all his observations; hence he left behind him a large amount of valuable writing.

He was engaged at the time of his death in a great work, his "Guiding Symptoms," and would to God he had been permitted to finish that work; but it was otherwise ordered. I am told by those who knew his habits, that every sentence in that work was studied over sometimes for hours, that his true meaning might be expressed. That he might lose no time, his writing desk and materials were brought close to the side of his couch, so that he could arise in the night light the lamp and continue his work. As for recreation and amusement, he knew little of either outside of his profession.

While a subject of the Saxon Government, he was commissioned to make collections as a naturalist in Surinam, South America. In the course of this study he found facts illustrating the truth of Homœopathy, and gave account of them to a Homœopathic journal in Germany. His Government objected to this work as heterodox. Dr. Hering thought he ought not to be controlled in any respect in the service of scientific truth. Upon the instant he resigned his commission and sought a free land, where his thoughts or the expression of them for the advancement of his race would not be controlled. He found such freedom in this country.

This showed his noble independence of character, and his earnest search and love of truth, which would not permit him to weigh against her a social position and a money consideration. He sought this new world to work and plow the field the providence of God assigned to him, with gifts to carry out fully and nobly his work, ere he was called away to be set in the heavens by the side of Hahnemann, Bœnninghausen, Staff and Jahr—a galaxy whose light will continue when the things of this earth and its monuments of brass and stone have crumbled.

Is it not wise and right that we should look into the sheaves of the rich harvest garnered by our late beloved colleague, for our own instruction, and that we should examine into the principles that govern him in the profession and practice to which he devoted his life, and in which he stood out so eminently the acknowledged leader?

Dr. Hering made this the essential point of doctrine and practice; to cure the sick easily and permanently, by medicines capable of themselves of producing in a healthy person morbid symptoms similar to those of the sick. He sought no other cure, nor recognized it as one, unless it was under the law proclaimed by Hahnemann. He sought no palliation, except under this law, believing that it hindered and endangered a perfect cure. He believed that the morbid condition of tissues and organs is the result of the dynamic disturbance, and not the cause of the disease. He was therefore a Vitalist—believing disease to be the disturbance of the vital force, and its equalization the state of health. He believed that the totality of symptoms, subjective and objective, is the only indication for the choice of a remedy. He did not believe that prescribing on the pathological states, nor diagnosis where the vital powers were tending to those states, was sufficient to effect a cure. The symptoms in their totality alone were the only guide for a cure to him.

He believed that the only proper way to ascertain the disturbing properties of medicine on the vital force is to prove them on the healthy; that thereby only the true expression of that disturbance can be observed. And he believed that, in order to obtain and secure the highest curative results, medicines must be administered singly and in a dose just sufficient to cure, because he knew that all action is followed by reaction: (there is no exception to this law) that all action on the vital powers is by an inherent law followed sooner or later by reaction which terminates in cure and health. Hence an overdose must by its intensity of action delay or prevent reaction and cure.

I remember on a certain occasion early in my practice, I told Dr. Hering of my suffering. He asked me the remedy I had taken, and seemed to think it well chosen. He then asked the dilution. I told him the third. "Ah;" said he, "you have stopped it, but perhaps not made a cure." He shook his head and seemed much disappointed. He said no more; but he caused me to reflect that it might well be so — that I had thrown an obstacle, before the diverted vital force — that I had stayed its forward movement by a shock that injured its reactive power— as a bowlder thrown before a carriage wheel in motion stops it, but cripples the wheel.

Dr. Hering believed that when he produced the impression at the right point, and in the right direction, the force mustbe permitted to be exhausted; therefore he waited. Shorter or longer the time he waited, his eyes wide open, and his observation on a stretch, looking for that action which is to end in equalization.

Dr. Constantine Hering was a true Homœopathist. He believed in that law and lived up to it. He believed that the highest results in his art were obtained by close individualization alone, not by generalization. I loved him for his simplicity and directness of character; for his large and brilliant enquiry after truth, and for his resting on principles derived from a patient examination of facts.

He enriched our Materia Medica by his severe labors. I will not name the many remedies he has proven, arranged and published. You know them all. The diligent student of our Materia Medica must have observed how full, exact and characteristic were the medicines proved and arranged by Hahnemann. Just so were the provings and arrangements by Dr. Hering equally clear, full, exact and characteristic. He took his great master, Hahnemann, as his model, and we only hope that those who may have the direction of arranging and publishing his writings, will give them to us just as he set them down. Then we shall feel that the seal of reliability is placed upon them.

When some patient astronomer who night after night has been watching the stars, brings to light some unknown planet, to do him honor the new-born world is called after his name and the discoverer is never to be forgotten. If the astronomer is worthy of this distinction, what shall we say of the man who brings to light a new remedial agent to relieve suffering humanity, ward off death, and bring back health? He, methinks, has done a greater work. And so the great discoverer of Lachesis will be gratefully remembered by those who know how to apply this remedy in all its varied forms, for which in the provings he suffered. And his only suffering was from the seal set by Lachesis, from which he never wholly recovered. That suffering was a crown of glory to him.

Constantine Hering showed in his death his medical principles, and showed that if the Homœopathic law, the law proclaimed by Hahnemann, was followed, a man would live longer and die easier than under any other practice; for he that is filled with disturbing drugs must die as the hunted fox, torn and rent by the bloody mouths of a pack of hounds. But he that follows the practice of our beloved colleague will have sleep rather than death. The forces equalized, he has rest. He ceases to exist by the withdrawal of his life by the giver of life; as some locomotive running smoothly upon the track, after exhausting her fuel, slows down and stops — not thrown from the rails by broken machinery, and rushing to ruin with terrible violence.

At six o'clock in the evening he made his last prescription to a patient, observing to his wife with great animation and interest that this patient had been prescribed for by many physicians, and he believed he should cure him. Then he went, as he was accustomed, to take his evening meal with his family, which he greatly enjoyed in that social circle under an arbor in his garden. At eight o'clock, the meal being over, Dr. Hering said he would retire to his study and his couch. His devoted wife went with him to aid him in preparing for bed. He said to her: "I believe I shall sleep." She left him to his repose. At nine he touched his bell which summoned her at once to his side. He remarked that his breathing was embarrassed, accompanied by constant yawning. He asked her to get a book in his office that he might examine this symptom. She did as directed; but being alarmed sent for a physician. I believe he selected the remedy and laid down to sleep. In a short time, without pain, without a struggle, he passed into that sleep which knows no waking—and the great physician demonstrated the benign, gentle, but controlling influence of the action of the great law to which he devoted his life. Thus died Constantine Hering, dear to Homœopathy, and to be forever honored by its true practitioners.

